# Biocides Used as Additives to Biodiesels and Their Risks to the Environment and Public Health: A Review

**DOI:** 10.3390/molecules23102698

**Published:** 2018-10-19

**Authors:** Glécia V. S. Luz, Breno A. S. M. Sousa, Adevilton V. Guedes, Cristine C. Barreto, Lourdes M. Brasil

**Affiliations:** 1 Postgraduate Program in Biomedical Engineering, Campus Gama (FGA), University of Brasília (UnB), Brasília 72.444-240, Brazil; lmbrasil@gmail.com; 2 Nanotechnology Laboratory, University of Brasília at Gama (NANOTEC-FGA/UnB), Brasília 72.444-240, Brazil; brenomarinhos@gmail.com (B.A.S.M.S.); adevilton.seq@gmail.com (A.V.G.); 3 Biotechnology Laboratory, Catholic University of Brasília, Brasília 70790-160, Brazil; criscbarreto@gmail.com

**Keywords:** biodiesel, additives, biocide, toxicity, public health

## Abstract

One of the advantages of using biodiesel and its blends with diesel oil is the lower levels of emissions of particulate matter, sulfur dioxide, carbon monoxide, among others, making it less harmful to the environment and to humans. However, this biofuel is susceptible to microbial contamination and biodeterioration. In this sense, studies on the use of effective low toxicity biocides are being carried out, and this work aims to present the latest information (2008–2018) available in the scientific databases, on the use of biocides in biodiesel, mainly concerning their toxicity to the environment and public health. The results showed that in relation to the control of microbial contamination, the current scenario is limited, with seven publications, in which the most studied additives were isothiazolinones, oxazolidines, thiocyanates, morpholines, oxaborinanes, thiocarbamates and phenolic antioxidants. Studies regarding direct experiments with humans have not been found, showing the need for more studies in this area, since the potential growth of biodiesel production and consumption in the world is evident. Thus, there are need for more studies on antimicrobial products for use in biodiesel, with good broad-spectrum activity (bactericidal and fungicidal), and further toxicological tests to ensure no or little impact on the environment.

## 1. Introduction

Biodiesels are composed of monoalkyl esters of fatty acids extracted from vegetable and microbial oils or animal fat. Alternative fuels based on biomass are achieving optimum results due to its efficiency and effectiveness. Compared to fossil fuels, biodiesel feedstocks are more accessible, renewable and economically viable [1].

Some studies on these fuels’ life cycle have shown that biodiesels release-less CO_2_ into the environment than diesel, a petroleum-derived compound. Therefore, biofuels contribute to the reduction of greenhouse gases. Also, the combustion emissions of biodiesel have lower levels of particulate matter, polyaromatic hydrocarbons, sulfur dioxide, carbon monoxide, aldehydes and ketones than fossil fuels, thus making it less harmful to humans. A practical concern for biodiesels is that the oil-like component, in the presence of water, provides a fertile environment for microbial growth, leading to the contamination of stored fuels, pipelines and compromising the characteristic fuel performance [2,3].

One can say that fostering biodiesel use could mitigate environmental issues, which is of a major importance in the world today [1,4]. However, biodiesel blends are highly susceptible to microbial contamination due to their hygroscopic capacity and its structure, predominantly composed by eight different methyl esterified fatty acids [5,6,7,8]. As a result, the contamination negatively affects product quality, compromises system and engine performance, as well as contributes to the corrosion of storage tanks [9]. Due to the practicality in the biodeterioration of biodiesels, this review focuses on the effective use of additives with low toxicity [10].

In this perspective, a biocide is added to biodiesel to inhibit or stop microbial growth; however, there is a lack of literature on the infallibility of its isolated use, in addition to a lack of research and publications analyzing the dangers of toxicity to the environment and society of the addition of the additive packages in the biofuel [11,12].

Antimicrobial pesticides, also known as microbicides or biocides, are chemicals used to eradicate microbes that contaminate fuel systems. Microbicides can be classified as bactericides or fungicides according to the target organism: bacteria or fungi, respectively. Other types of microbicides can also function as broad spectrum antimicrobial pesticides because they are effective against both bacteria and fungi and algae [10]. Furthermore, biocides can also be categorized based on their fuel and water solubility as water soluble, fuel soluble or dual soluble.

The 2009 World Health Organization (WHO) Recommended Classification Of Pesticides By Hazard and Guidelines to Classification document [13] sets out a classification system to differentiate between the greater and lesser hazardous forms of pesticides based on acute risk to human health, i.e., the risk of single or multiple exposures within a short period of time. The document considers the toxicity of the active substance and refers to methods for the classification of formulations. However, data on biocide selection for fuel use should be based on objective treatments and proper regulations, which may vary from country to country.

For example, in the United States the use of biocides is regulated by the United States Environmental Protection Agency (USEPA) which requires prior approval from the USEPA Office of Pesticides Programs (40 CFR 152) [14,15]. American Standards ASTM D 6469-17 provides a guideline for microbial contamination in fuels and fuel systems, which includes strategies for controlling microbial growth [14]. In Europe, the antimicrobial pesticides are regulated by the Biocidal Products Directive in the European Economic Union, Regulation (EU) No 528/2012 [16]. In Brazil, it is no longer necessary for these additives to be registered in the National Petroleum Agency (ANP), which aims to simplify administrative processes and reduce regulatory costs, intended to stimulate competition and offer of products in the market. The ANP considers the registration of additives for automotive fuel to be unnecessary, since the Agency is already engaged in quality assurance of fuels (additives or not), which is provided through monitoring programs directed to protect the interests of consumers [17].

Regarding the concentration levels of biocides found in biodiesel and diesel-biodiesel blends, which are already contaminated systems, the recommendation is the application of 1000 ppm [18,19]. Preferably, the dosage can be, for example, 100 ppm [19], 200 ppm [20,21] or 400 ppm [18]; in which the concentration variation may follow the product’s distributor’s guidelines.

Biocides are products with a large range of components and chemical structures, and can be characterized as oxidants (ozone, hydrogen peroxide, chlorinated compounds) and non-oxidants (sulfur compounds, tin compounds, isothiazolones, copper salts, etc), while the oxidants aim at oxidizing the microbial cells’ components, the non-oxidants interfere with metabolism and cellular disintegration [9].

A biocide’s performance depends on the different cell types of the microorganisms that are exposed to it. Isothiazolinones, for example, initially act with their biostatic properties and promote the loss of the thiol group (SH) binding of the polymer chains of proteins that are necessary in cellular structure and functioning [21].

Several approaches have been used to investigate biocides′ ability to degrade fuel microorganisms, but there is no standard protocol for biodiesel degradation testing, although it is considered to be a particularly vulnerable fuel for microbial deterioration [9,22,23].

Therefore, the objective of this work is to present the latest scientific information available on the use of biocides in biodiesel, and to report relevant work on their toxicity to the environment and public health.

## 2. World Biodiesel Production

The advantage of biodiesels, if compared to conventional fuels, is its lower levels of pollution in the refining, production and use of the product which, in turn, has demonstrated to be harmless to the environment [1,24]. Despite this great advantage, biodiesels have higher production costs when compared to other fuels [24]. Their high large-scale production cost is due to the need for specialized professionals and sophisticated equipment and infrastructure. Additionally, the synthesis involved in the production process requires specialized knowledge in multiples areas, such as organic chemistry, biochemistry, physical chemistry, safety, reagent and waste toxicity [24]. The synthesis process of biofuels can use methanol, an extremely poisonous chemical, as well as strong bases, such as sodium hydroxide (NaOH), which are also highly toxic and corrosive [24].

**Figure 1:**
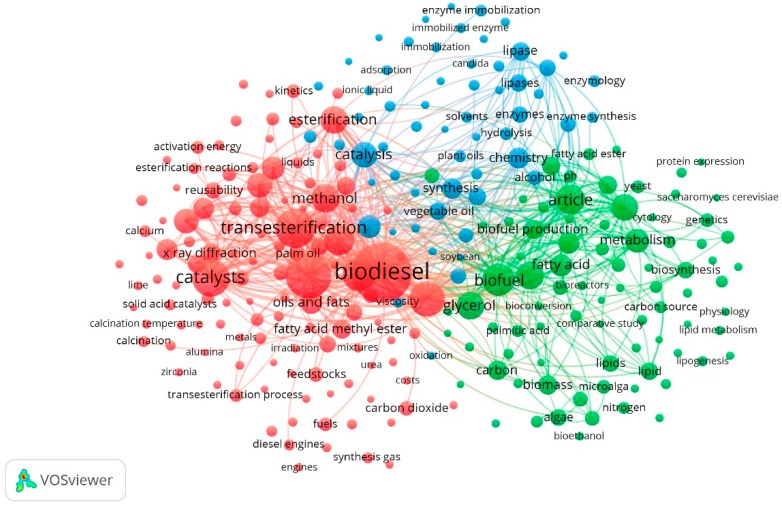
The 1770 research papers found in the Scopus database, from 2014 to 2018, with the string “Biodiesel AND Synthesis”; its recurrences and connectivity between specific terms (analysis in VOSviewer software 1.6.8 (2018)). Source: Scopus [28].

The transesterification technique is the most common method used to produce biodiesel, increasing the reaction of the triglycerides present in vegetable oils and animal fats with alcohol in the presence of a catalyst [25,26,27]. Potassium hydroxide (KOH) and sodium hydroxide (NaOH) are used as alkaline catalysts to produce biodiesel and represent one of the most conventional ways for this process. Thus, these reagents are usually inserted in the synthesis routes in industrial and commercial scale at a low cost [2,27]. Using the search string “TITLE-ABS-KEY (Biodiesel AND Synthesis) and PUBYEAR > 2013”, a total of 3294 papers published in the Scopus database until 25 August 2018 were found. From this set, 1770 were published only in the last five years (from 2014 to 2018). Regarding these 1770 papers, the map in Figure 1 shows the repetition of the search strings (at least 20 times) in their titles, keywords and abstracts.

**Figure 2:**
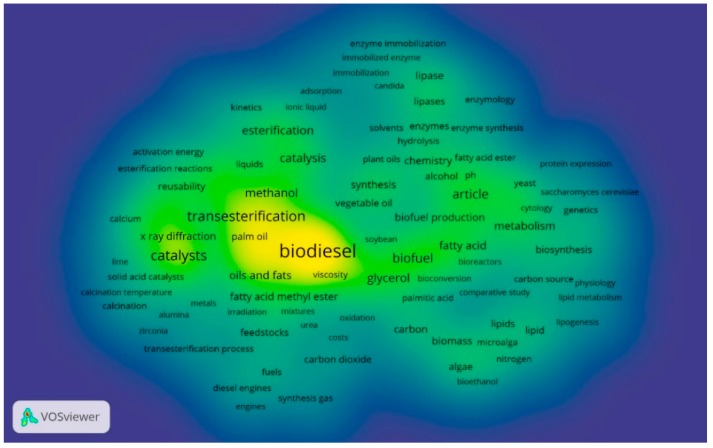
Data with a research database based on Scopus, between the years 2014–2018, with the string “Biodiesel AND Synthesis” found recurrences between specific terms. The clearer the color, the greater the number of recurrences of this term (analysis in software VOSviewer 1.6.8 (2018)). Source: Scopus [28].

There are three clusters with a total of 261 terms. The cluster associated with the biodiesel term is the one with the highest recurrence and it is highlighted in yellow in Figure 2.

According to Figure 1 and Figure 2, the most frequently used terms are catalysis [27,29], homogeneous catalyst [30], heterogeneous catalysis [31,32,33], biosynthesis [34], esterification [27,31,35], transesterification [36,37,38], enzyme synthesis [39,40], microalga. Additionally, the most exploited synthetic routes are by transesterification and esterification.

Different synthetic routes and catalysts are being used, because the most common synthesis implicates the execution of multiple processes, such as glycerol purification, pH solution neutralization, biodiesel washing-drying, and wastewater treatment [27,41]. Furthermore, the application of alkali catalysts is limited to the acid number of the waste lipid source. It is known that, when the acid number is too high it is preferable to use another catalyst class in order to avoid an excess of soap formation in solution and low yields [27]. Therefore, enzyme-catalyzed transesterification, as well as biosynthesis, could be both promising alternatives for the synthesis of this biofuel.

The worldwide production of biodiesel is lower than that of ethanol. However, while ethanol is produced mostly in Brazil and in the USA, biodiesel is manufactured in multiple countries around the world, each one maintaining their own legislations and regulatory procedures, as well as different business strategies. This worldwide production facilitated the entry of the product on the world’s commodity list, favoring the development of this new market on a global scale [41,42].

In Brazil, Law N°. 11097/2005 (National Program for the Production and Use of Biodiesel) determined the compulsory use of a minimum percentage (5%) of biodiesel in diesel oil (B5). This law lasted eight years [43], until 2013, when a new resolution 647/2014 [44] established a compulsory percentage of 7% (B7) of biodiesel in diesel oil. The National Agency of Petroleum, Natural Gas and Biofuels (*Agência Nacional do Petróleo*, *Gás Natural e Biocombustíveis*—ANP) is the entity responsible for this resolution and many others published in subsequent years, which have raised the levels of biodiesel applied to fuels in Brazil. In 2018 the percentage allowed was changed to 10% (B10) [45] and the Law N° 13.033 establishes that this number may increase up to the limit of 27.5% [46]. In addition to the standardized minimum values, other percentages such as B20 (20%), B50 (50%) and B100 (100%) are also used in researches. In the international scenario, these percentages are not different, and, in some countries, the percentages are even higher.

**Figure 3:**
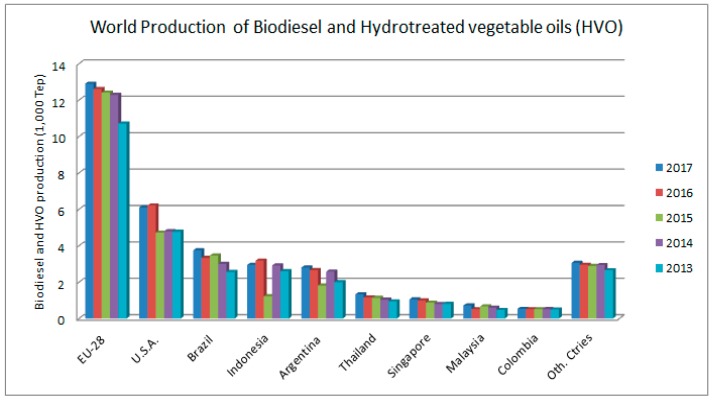
Data about world production of Biodiesel and hydrotreated vegetable oils (HVO). Source: OilWorld (2018) [47].

Figure 3 shows the growth of biodiesel production and some of the biggest producers: the European Union (EU), the United States of America (USA), and Brazil. Biodiesel has the potential to promote economic growth and ecological awareness, by replacing the use of fossil oil for renewable fuels.

While the EU uses rapeseed to produce biodiesel, in the Americas soybeans are the preferred raw material. The main biodiesel producers nowadays are the U.S.A., Brazil and Argentina. Southeast Asia has achieved greater prominence in the biodiesel market. In Indonesia and Malaysia, which are palm oil-producing countries, biodiesel production is growing continuously, controlled by structural oversupply and price pressure in vegetable oil markets [48]. This review shows that researches on biodiesel synthesis have increased, but a main concern is what can be said about its stability, the microbiological contamination, and the consequences of the use of biocides in biodiesel on the environment. This is the question that this paper intends to answer.

## 3. Materials and Methods

### 3.1. Eligibility Criteria

This work considered original peer-reviewed articles, published in English, about biocides used as additives to biodiesel, with special attention to their toxicity for the environment and public health. The results were organized in subtopics to better represent the subjects covered by this research, considering the period between 2008 to 2018.

### 3.2. Data Items

A broad literature research was conducted in March 2018 using the PubMed/MEDLINE, Scielo, LILACS/BVS, IEEE, ACM, ScienceDirect and Scopus databases. Additional searches using the Cochrane Library, Web Portal of Articles from CAPES and cross-referencing with Web of Science were used to broaden the initial search results. Table 1 describes the keywords applied for each research database.

### 3.3. Study Selection and Data Collection Process

Four reviewers (G.V.S.L., B.A.S.M., A.V.G. and L.M.B.) independently evaluated all relevant articles extracted from the literature research. The full text of each article was obtained and further screened for selection if it showed relevance to the predefined topics and/or subtopics. Five reviewers (G.V.S.L., B.A.S.M., A.V.G., C.C.B., and L.M.B.) worked on organizing the results and the overall data layout. All reviewers worked on writing the “Results” and “Discussion” sections. The research was conducted according to the stages described in SLR Model [49].

### 3.4. Data Items

The following items were extracted from the studies: ‘Type of Study’, ‘Objective of work’, ‘Biocide (s) cited at work’, ‘Type of study carried out with the biocide’, ‘Does this study provide data on the toxicity of biocides in biodiesel?’ and ‘Work Result(s)’.

## 4. Results

**Figure 4:**
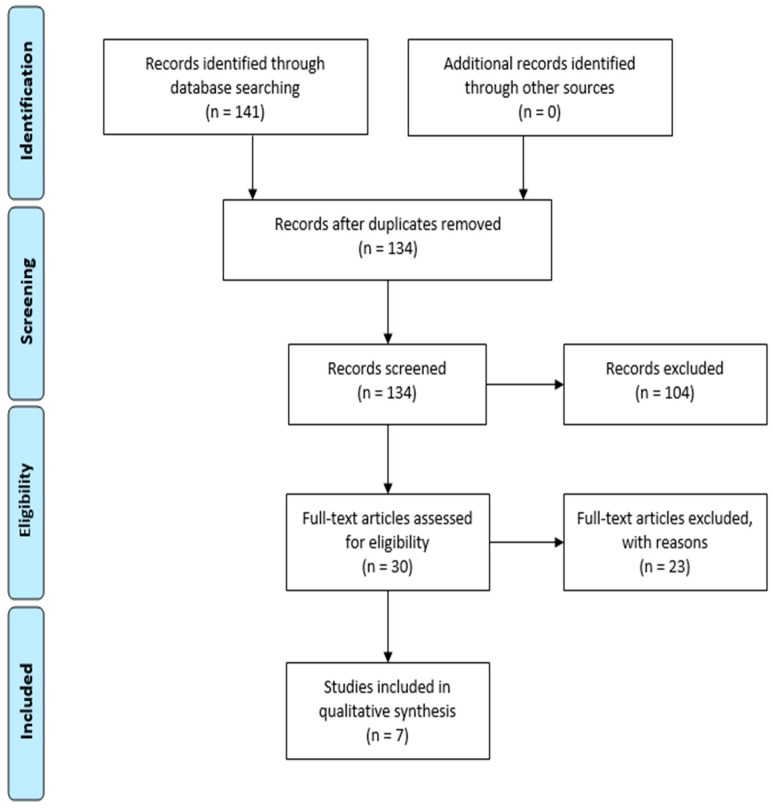
General work used to select relevant research studies (PRISMA flow diagram).

Initial investigations using the keywords revealed 141 scientific papers. Table 2 summarizes the findings, which shows a large amount of scientific works in the databases of IEEE, “*Periódicos* CAPES”, and in ScienceDirect. No publications were found in the other databases. Once duplicates were removed, 134 studies remained and matched the inclusion criteria. These studies comprise the selected articles discussed in this review. The general workflow used to choose relevant research studies is depicted in Figure 4. Table 3 includes the information for each selected research study, such as: authors, objectives, biocides used, type of study, data about the toxicity and overall conclusions.

## 5. Discussion

**Figure 5:**
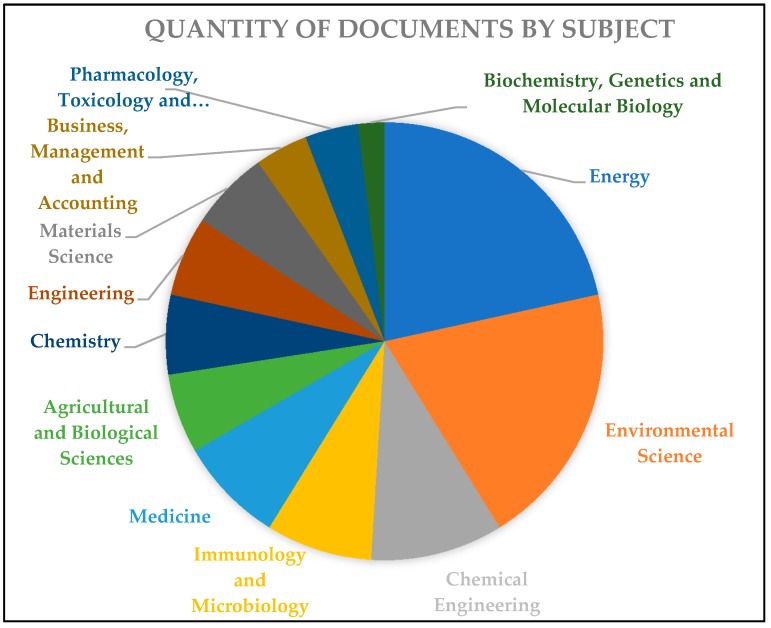
Quantity of documents found by subject area in the period of 2008–2018, using “biodiesel AND biocide” string for research in database Scopus [28].

By conducting research on the Scopus database, using the keywords “biodiesel AND biocide”, it was shown that the studies of these quantities are multidisciplinary (Figure 5). The graph below shows that the concentrations areas of these researches and their respective publications are proportionally more located in, for example, engineering, chemistry and agriculture—which is the sector focused on the reduction of biodiesel toxicity, mainly with the use of biocides with fungal, bacterial and organic material inhibitory functions.

**Figure 6:**
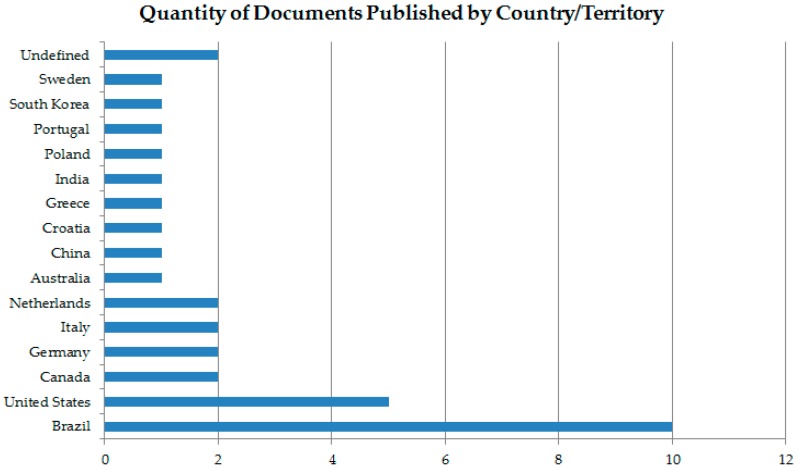
Quantity of documents published by country/territory in the period of 2008–2018, using “biodiesel AND biocide” string for research in database Scopus [28].

Looking at Figure 6 and Table 4, there is a larger amount of publications on “biodiesel AND biocide” (2008–2018 period) in countries such as Brazil, United States of America (USA), Canada, Germany, Italy, among others. In Table 4, there are 9 publications on “Biodiesel AND Biocide”. In general, all affiliations have around two references, which shows the low number of published works in the area. However, it is possible to see the interaction between countries that produce and/or consume this type of biofuel, to find solutions to its instability. Furthermore, it is possible to conclude that science needs to expand studies in this field, since this product has presented several benefits, such as low toxicity and the improvement of life quality and reduction of gas emission. Is this case, it was noted that in a small ranking of publications per country, Brazil takes the lead (Figure 6 and Table 4).

Studies involving experiments with humans have not been found. It must be added that the two primary pillars of microbial contamination control are: prevention and remediation. Prevention includes system design, water removal and good cradle-to-grave product stewardship.

**Figure 7:**
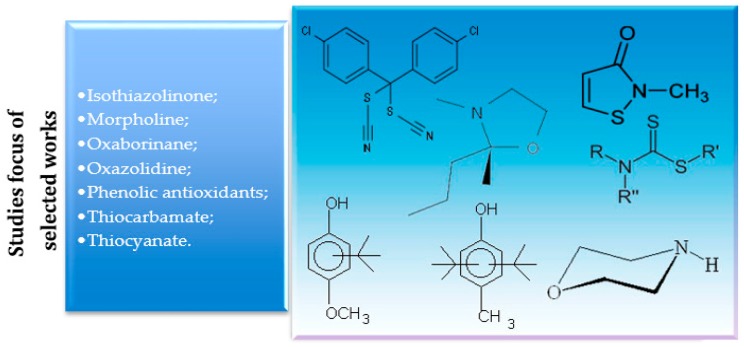
Biocides cited in the seven works selected about biocides, biodiesel and toxicity published in the period of 2008–2018.

In the case of the research that restricts the selection of the scientific works published on the context of toxicity, Table 3 shows that the seven papers found were focused on the studies of substances such as isothiazolinones, oxazolidines, thiocyanates, morpholines, oxaborinanes, thiocarbamates and phenolic antioxidants, as it can be seen in Figure 7.

The seven works showed microorganisms tests in biodiesel and biodiesel blends. Poon et al. [1] related AM tests system on toxicity for the biocides. Their work reported that the order of greatest toxicity would be to CMIT and MDC, decreasing to MIT, and showing DMAD as non-toxic. The authors suggested further studies about 5-chloro-2-methyl-4-isothiazolin-3-one (CMIT), because when it is added in biodiesel it might provoke respiratory complications. Pelletier et al. [58] studied MIT/CMIT in rats, and on recommended levels for the microbial treatment in fuels, in this accidentally ingested of these ones is not expected to express a significant health risk. Other authors who cited the isothiazolinone biocide in their research were: Passman [9], Zimmer [6] and Bautista et al. [52], indicating the considerable interest in this biocide for use in biodiesel and its mixtures.

Oxazolidines also appear rather frequently in the selected studies and are registered with regulatory organizations to be used as a biocide in fuels. The authors who included information regarding this type of biocide in their works are: Passman [9], Zimmer [6], Bautista et al. [52] and Zimmer et al. [18]. For example, in Bautista et al.’s work [52], ten biocides were studied in samples of diesel oil in fuel storage tanks. The authors observed that the best results were related to the water-soluble biocides, notably those with an oxazolidine group. In addition, Zimmer et al. [18] studied the effectiveness of a multifunctional additive containing a MBO biocide as 50% of its formulation (AM-MBO50) for microbial control contamination under simulated storage conditions. The results showed that the product is efficient for the microbiological control without affecting the properties of the biodiesel. However, it should be considered that when comparing laboratory scale and field tests, the results might suffer variations, with monitoring and possible adjustments thus being of vital importance.

Toxicity studies in rats with only biodiesel samples, i.e., without additives, were performed by Finch et al. [59,60]. In this study, the effects associated to the exposure were limited to the lung, and showed modest adverse effects at the highest level of exposure, and none other than the expected response of physiological macrophages to repeated particle exposure at the intermediate level. Also without adding additives, the study by Brito et al. [61] evaluated the cardiovascular and inflammatory toxicity of particles emitted from the combustion of diesel and biodiesel, evidencing greater biodiesel toxicity than diesel because it promoted cardiovascular alterations and pulmonary and systemic inflammation. Mehus et al. [62] investigated the human health effects of comparative acute health associated with exposures to diesel and diesel-biodiesel fuel blend emissions (75–25%). The results showed that the use of this diesel-biodiesel blend decreased the exposure to particulate diesel respirable material and some acute effects associated with health, although lung and systemic inflammation were not reduced in comparison with the use of diesel. The work of Imdadul et al. [63] provided information on the impact of the following additives and its effects on performance and emission: oxygenated, metal-based, cetane number improver, ignition promoter, lubricant and antioxidant. The authors concluded that the characteristics of combustion and exhaust emissions have obtained better results through the introduction of additives in diesel and biodiesel blends.

This review didn’t evaluate the use of biodiesel + biocides after emission from a diesel engine, as described in Table 3. Nevertheless, if more searches in the databases are conducted, it might be possible to find studies evaluating, for example, an additive package (antioxidant + pour-point depressant + biocide) [21,64]. Luz et al. studied biocides belonging to the isothiazolinone (200 ppm) group. The authors reported that the additive package’s addition did not increase greenhouse gases and CO_2_ emissions; if anything, it helped minimize HC emissions in the environment, especially in mixtures with 20% biodiesel in their composition. However, more detailed analyzes of the exhaust gases from the engine were not performed. Overall, the findings regarding biocide studies in biodiesel, including exhaust emission data, are still scarce, which indicates a scientific gap that might motivate future research.

## 6. Conclusions

Biodiesel is a great fuel alternative because it is obtained from renewable sources and is economically profitable. However, biodiesel blends are susceptible to microbial contamination due to their hygroscopic capacity and their methyl esterified fatty acids composition. The ease of oxidation and biodeterioration of this biofuel has prompted research on the use of effective additives with low toxicity, avoiding the decrease of the quality of the product, as well as complications in system and engine performance, and corrosion of storage tanks and pipelines.

This review did not find any research between the years of 2008 to 2018, considering the inclusion and exclusion criteria, that conducted direct experiments with humans. Out of the 141 potential works, only seven which contained information on the use of biocides and microorganism tests in biodiesel and biodiesel blends were selected. These studies focused on biocides, such as isothiazolinones, oxazolidines, thiocyanates, morpholines, oxaborinanes, thiocarbamates and phenolic antioxidants. The need for further studies in the field was also noted, and it can be solved mainly by applying laboratory tests in large scale tests, in order to determine possible influences on the final results of microbial control of the environment, and contribute to the topic of toxicity to the environment and public health.

There are few publications showing the importance of controlling microbial contamination, which began at the end of the 19th century, in view of the evident future prospect of growth in the use and production of this biofuel in the world. The remediation tactics, such as biocide treatment, should be driven by knowledge of the nature of the studied infected systems, regulatory constraints and technical considerations. Thereby, in the search for new antimicrobial products for biodiesel, biocides must offer many things, amongst them good broad-spectrum (bactericidal and fungicidal) activity and environmental tests resulting in lower negative impacts.

## Figures and Tables

**Table 1:** Strings used for searches in databases.

Research Base	Strings
PubMed/MEDLINE	biodiesel AND additive AND biocide AND toxicity
Scielo	biodiesel AND additive AND biocide AND toxicity
LILACS/BVS	biodiesel [Palavras] and additive [Palavras] and biocide [Palavras] and toxicity [Palavras]
IEEE	biodiesel AND additive AND biocide AND toxicity
ACM	biodiesel AND additive AND biocide AND toxicity
ScienceDirect	biodiesel AND additive AND biocide AND toxicity
Cochrane Library	biodiesel AND additive AND biocide AND toxicity
Periódicos CAPES	biodiesel AND additive AND biocide AND toxicity
Web of Science	TOPIC: (biodiesel) *AND* TOPIC: (additive) *AND* TOPIC: (biocide) *AND* TOPIC: (toxicity)
Scopus	TITLE-ABS-KEY (*biodiesel* AND *additive* AND *biocide* AND *toxicity*)

**Table 2:** Number of scientific papers found during searches in databases.

Research Base	Number of Scientific Papers Found
PubMed/MEDLINE	0
Scielo	0
LILACS/BVS	0
IEEE	32
ACM	0
ScienceDirect	94
Cochrane Library	0
Periódicos CAPES	15
Web of Science	0
Scopus	0

**Table 3:** Data obtained from selected scientific works from databases.

Ref. Number	Authors (year)	Type of Study	Objective of Work	Biocide(s) Cited in the Work	Type of Study Carried out with the Biocide	Does this Study Provide Data on the Toxicity of Biocides in Biodiesel?	Work Result(s)
[1]	Poon et al. (2011)	Article	This study is on the effects of 5-chloro-2-methyl-4-isothiazolin-3-one (CMIT).	5-Chloro-2-methyl-4-isothiazolin-3-one (CMIT), 2-methyl-4-isothazolin-3-one (MIT), methylene dithiocyanate (MDC) and dimethyl acetylenedicarboxylate (DMAD)	Studies about organic biocides using freshly isolated rat alveolar macrophages (AM) and NR8383 cell line.	Toxicity studies four types of biocides.	The 50% inhibition concentration (LC_50_) for CMIT was 0.002–0.004 mM for both cellular functions. With the AM testing system, the toxicity for the biocides were CMIT = MDC > MIT > DMAD. The authors suggested that CMIT added in biodiesel might provoke respiratory impairment, and more studies using animal subjects are warranted.
[9]	Passman (2013)	Review	This work relates informations about the factors involved in fuel to the fuel system biodeterioration.	Diiodomethyl-*p*-tolylsulfone; Ethylene glycol monomethyl ether (EGME); Diethylene glycol monomethyl ether (DiEGME); Triethylene glycol monomethyl ether (TriEGME-M); dioxaborinane blend; Isothazolinone blend; morpholine-dinitromorphiline blend; 3,3’-methylenebis(5-methyloxazolidine) (MBO; 95–100% a.i.)	Studies about remediation strategies with biocide treatment.	The work provides information on antimicrobial studies, among others.	One of the results is that the fuel treatment represents a tiny fraction (<0.1%; Passman, 1995 [50]) of the total industrial microbiocides market. Although the use of fuel-treatment microbiocides is likely to increase, new chemistries are unlikely to emerge.
[35]	Zimmer (2014)	Master Thesis	This work was made to select antimicrobials to be used in the control of microbial contamination of diesel/biodiesel.	Oxazolidine, isothiazolone and morpholine.	Studies on microbial control and microbial contamination.	The work only provides data regarding microorganisms.	It was found that an additive containing 50% oxazolidine in its formulation was effective for the preventive control of microbial contamination in B10 mixtures. In addition, testing containing isothiazolones or morpholines may be a good option for corrective treatments. The toxicity results show that both water, which was in contact with the treated (with the additive) and untreated, showed toxicity to the organisms used. However, the water that was in contact with the biocidal additive fuel showed high acute toxicity for both test organisms studied.
[51]	Pelletier et al. (2014)	Article	This study reported the health effects of CMIT/MIT ingestion in rats to give information about the potential health risks that may arise from the grow up in the use of biocides in biodiesels or biodiesel blends.	2-Methyl-4-isothiazolin-3-one (MIT), 5-chloro-2-methyl-4-isothiazolin-3-one (CMIT).	Randomized study in male and female rats.	An oral dose study was conducted to assess a potential risk arising from ingestion of isothiazolinone biocides in biodiesels.	Based on recommended levels of biocides for the microbial treatment in fuels, CMIT/MIT contained in this accidentally ingested biofuel is not expected to express a significant health risk.
[52]	Bautista et al. (2016)	Article	Compare and evaluate the efficiency of several chemical and physical treatments on the growth of microorganisms found in real samples of diesel fuel from different storage tanks from petrol stations in Spain	Isothiazolone; oxazolidine; thiocyanate; thiocarbamate; morpholine; oxaborinane.	Studies about organic biocides.	Studies on remediation strategies with biocide treatment.	According to the results, water-soluble biocides (especially B2 with oxazolidine group in the active compound) showed higher performance in controlling bacterial growth in the studied diesel fuel storage tanks. However, the effectiveness of biocides very much relies on biodiversity and physicochemical properties of the medium. In order to control growth of microorganisms in oil storage tanks, some preliminary studies, (on a case-to-case basis) on the microbial population and physicochemical characteristics inside tanks and in the surrounding area where the tank is located (soil composition, environmental conditions, climate, etc.) must be performed.
[48]	Dodos; Tsesmeli, and Zannikos (2017)	Article	This study aimed to investigate the effect of phenolic type antioxidants on the microbial stability of biodiesel fuel, along with their relative efficiency to improve the oxidation and storage stability.	Ten commercially available phenolic compounds, either of synthetic or natural origin.	Studies about antimicrobial properties.	The work only provides data regarding microorganisms.	Overall, the results demonstrate that certain phenolic antioxidants primarily added to biodiesel in order to improve oxidative stability could also provide a satisfactory level of antimicrobial protection at the same time. Although these substances do not necessarily possess biocidal properties, they appear as non-supportive to active biomass. This suggests that by properly selecting a FAME antioxidant agent, the microbial stability of bio-diesel can be upgraded up to a point.
[53]	Zimmer et al. (2017)	Article	The objective of this study was to assess the effectiveness of an additive multifunctional biocide to treat microbial contamination under simulated storage conditions.	3,3-methylenebis(5-methyloxazolidine) (MBO)	The tests were managed under two conditions: at lab-scale and in the field (real-world condition).	Experiments were carried out in the laboratory (lab-scale; 250 mL microcosms) and in the field (field-scale; 20 L tanks) under real-world conditions.	The lab-scale study showed that this product was able to inhibit biomass formation in the range of 40% to 60% during simulated fuel storage in the microcosms, at a 400 ppm concentration. In the field-scale study, the multifunctional additive at a 1000 ppm concentration showed a biocide action after 7 d in the tanks with low microbial contamination and a biostatic action in the tanks receiving microbial inoculum (high contamination).

**Table 4:** Quantity of documents published by affiliation in the period of 2008–2018, using “biodiesel AND biocide” string for research in database Scopus [28].

University	Publications	References
Universidade Federal do Rio Grande do Sul	4	[18,53,54,55]
Instituto Nacional de Tecnologia do Rio de Janeiro	3	[53,54,56]
Ipiranga Produtos Petróleo	2	[18,53]
Wageningen University and Research Centre	2	[51,57]
Universidade Federal de Viçosa	2	[51,57]
Health Canada	2	[1,58]
Consiglio Nazionale Delle Ricerche	2	[20,21]
Universidade de Brasília	2	[20,21]
Istituto Motori	2	[20,21]
